# Chimeric tumor modeling reveals role of partial PDL1 expression in resistance to virally induced immunotherapy

**DOI:** 10.1186/s40425-018-0496-6

**Published:** 2019-01-16

**Authors:** Mee Y. Bartee, Parker C. Dryja, Eric Bartee

**Affiliations:** Department of Microbiology and Immunology, Medical University of South Carolina, Basic Science Building Rm 208C, 173 Ashley Ave, Charleston, SC 29425 USA

**Keywords:** Immunotherapy, Oncolytic virotherapy, Myxoma virus, Tumor heterogeneity, PDL1

## Abstract

Expression of PDL1 on the surface of tumor cells can blunt the efficacy of many cancer immunotherapies. For example, our lab has previously shown that tumors derived from malignant cells incapable of expressing PDL1 are highly susceptible to immunotherapy induced by oncolytic virus treatment while tumors derived from PDL1 capable cells are highly resistant. In patient biopsies, however, expression of PDL1 on malignant cells is often not uniform with some cells expressing PDL1 while others do not. Importantly, how this partial PDL1 positivity influences the outcomes of immunotherapy remains largely unknown. In the current work, we expand on our previous findings by generating partially PDL1 positive tumors in immune competent animals and asking what percentage of tumor cells must express PDL1 for a tumor to become functionally resistant to oncolytic treatment. Our results indicate that the responsiveness of partially PDL1^+^ tumors correlates linearly with the percentage of PDL1 capable cells present at the initiation of treatment. Additionally, we observe that tumors which relapse after treatment display a significant increase in the numbers of PDL1 capable cells present suggesting that specific editing of mixed tumors might play a role in disease relapse. These data indicate that varying levels of PDL1 expression can play a significant role in the outcomes of oncolytic immunotherapy and challenges the concept that tumors should be viewed as simply PDL1^+^ or PDL1^−^.

**Electronic supplementary material:**

The online version of this article (10.1186/s40425-018-0496-6) contains supplementary material, which is available to authorized users.

## Introduction

Programmed cell death protein 1 (PD1) is an immune checkpoint protein which is rapidly expressed on T cells following their activation. Engagement of PD1 with its primary ligand, programmed death ligand 1 (PDL1), results in T cell dysfunction and eventual exhaustion [1, 2]. This pathway exists to maintain peripheral tolerance and prevent autoimmunity, however, overexpression of PDL1 within the tumor microenvironment is seen in a variety of malignancies and likely represents a major mechanism through which cancer escapes immune surveillance [3, 4]. While PDL1 can be found on a variety of cell types within the tumor microenvironment [5–10], expression is most frequently observed on malignant tumor cells themselves were it can range from completely PDL1^−^ to virtually 100% PDL1^+^ [11–15]. In patients whose tumor cells are PDL1 positive, this expression displays two distinct patterns [16]. The first, inducible expression, presents as a spatially separated PDL1^−^ core surrounded by PDL1^+^ cells and results from upregulation of PDL1 by IFNγ expressed from tumor infiltrating lymphocytes (TIL) [17, 18]. The second pattern, constitutive expression, presents as tumors which varying mixtures of both PDL1^+^ and PDL1^−^ cells and is caused by mutations, such as EGFR activation [19] or loss of PTEN function [20], within individual cells. Despite this complex intratumoral expression pattern, there is not a consensus on how many PDL1 expressing tumor cells are required to constitute a functionally PDL1^+^ tumor. Therefore, identifying how expression of PDL1 on specific percentages of tumor cells impacts the outcomes of immunotherapy, and what constitutes a functionally PDL1^+^ tumor, remain critical unanswered questions.

Oncolytic virotherapy (OV) is a form of localized immunotherapy in which injection of a cancer-tropic virus results in the generation of potent anti-tumor T cell responses [21, 22]. We have previously demonstrated that tumors which are incapable of expressing PDL1 on their malignant cells are highly susceptible to OV while those which express high levels of PDL1 are highly resistant [23]. In the current manuscript, we now expand on our previous findings by using a novel method of chimeric tumor modeling to generate partially PDL1^+^ tumors in immune competent animals and study how this partial PDL1 positivity impacts the outcomes of virally induced immunotherapy.

## Results

### Generation of PDL1-chimeric tumors

**Fig. 1:**
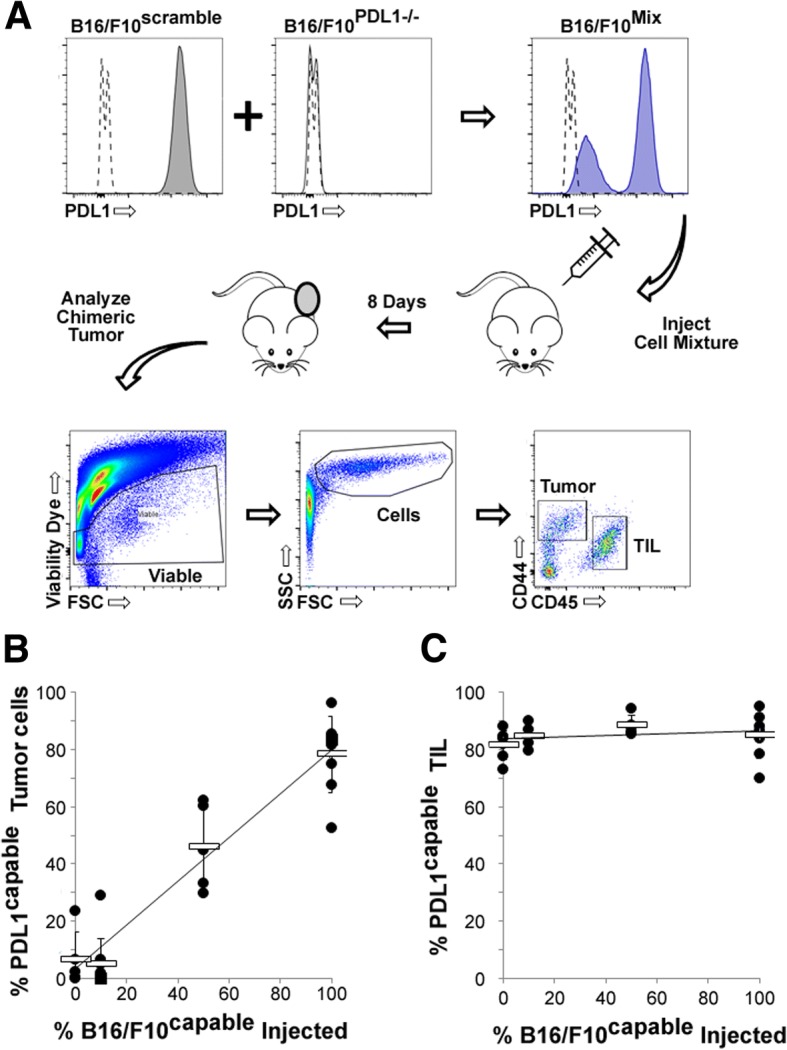
Generation of PDL1 Chimeric Tumors. **a** Schematic representation of generation and analysis of in vivo chimeric tumors. **b** Average percent of PDL1 capable tumor cells found in tumors initially generated from the indicated cellular makeup. Data represents summation of two independent experiments. **c** Average percent of PDL1 capable TIL found in tumors initially generated from the indicated cellular makeup. Data represents summation of two independent experiments

Our previous studies have demonstrated that the presence of PDL1 on tumor cells plays a significant role in determining the outcomes of MYXV-based OV ( [23] and Additional file 1: Figures S1 and S2). However, while PDL1 is expressed on a high percentage of malignant cells in most preclinical models [23], analysis of primary tumor biopsies has revealed a much more complex pattern of expression [12, 24]. In order to assess how this partial PDL1 positivity impacted oncolytic immunotherapy, we therefore used chimeric tumor modeling to generate tumors in vivo which contained various percentages of PDL1^+^ cells. B16/F10^scramble^ and B16/F10^PDL1−/−^ cells were mixed ex vivo at either a 1:1 or 1:9 ratio and the mixtures then injected into C57/B6 mice (Fig. 1a). As controls, pure cultures of either B16/F10^scramble^ or B16/F10^PDL1−/−^ cells were injected in parallel. To assess the success of this strategy at generating partially PDL1 positive tumors, animals were euthanized 8 days after injection and the capacity of tumor cells to express PDL1 was analyzed (Fig. 1b). Consistent with the formation of PDL1-uniform tumors, animals injected with pure cultures of B16/F10^scramble^ cells developed tumors which were capable of expressing PDL1 on the vast majority of malignant cells (78.2% PDL1 capable) while animals injected with pure cultures of B16/F10^PDL1−/−^ cells developed tumors which were virtually devoid of PDL1 expressing malignant cells (2.1% PDL1 capable). In contrast, animals injected with a 1:1 ratio of B16/F10^scramble^ to B16/F10^PDL1−/−^ cells developed tumors which were capable of expressing PDL1 on 46% of their tumor cells, while animals injected with a 1:9 ratio developed tumors capable of expressing PDL1 on 4.9% of their tumor cells(Fig. 1b). These data suggest that the tumors resulting from injection of mixtures of PDL1 capable and PDL1 deficient cells correlate linearly with the cellular input. Injection of different mixtures of PDL1 capable and PDL1 deficient cells did not impact the expression of PDL1 on tumor infiltrating lymphocytes (TIL) which were uniformly capable of expressing this protein regardless of the makeup of the injected malignant cells (Fig. 1c).

### Partial PDL1 positivity linearly impacts the outcomes of oncolytic immunotherapy

**Fig. 2:**
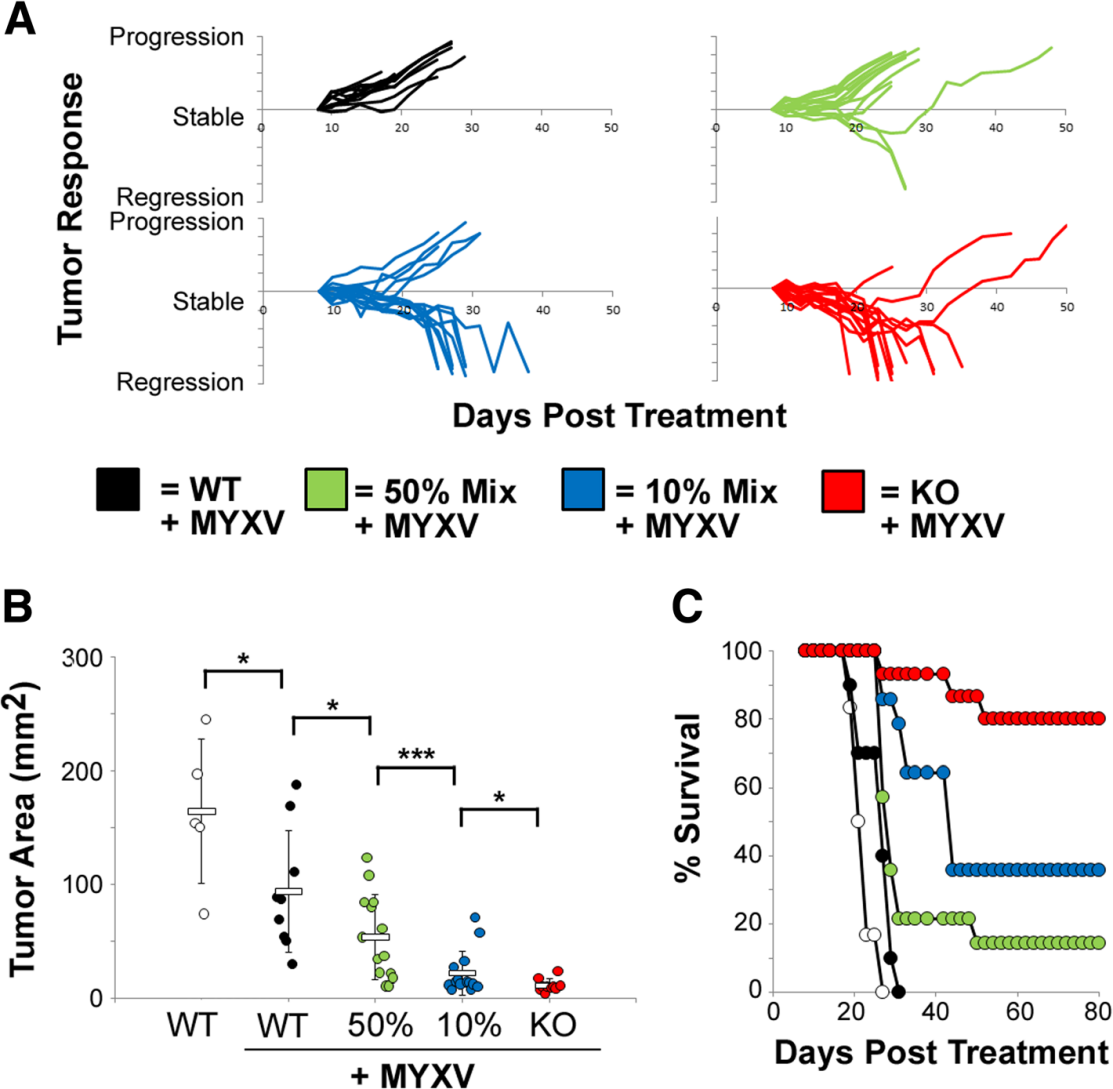
PDL1 Chimeric tumors are resistant to oncolytic therapy. **a-d** 4 × 10^5^ total cells comprising pure cultures of either B16/F10^scramble^ (WT: *n* = 10), or B16/F10^PDL1−/−^ (KO: *n* = 15), or either a 1:9 mix (10% *n* = 14), or a 1:1 mix (50% n = 14) of WT to KO cells were injected SQ into the left flank of syngeneic C57/B6 mice. 7, 9, and 11 days post tumor implantation; tumors were treated with IT injection of 1 × 10^7^ foci forming units of MYXV. Tumors were then monitored every other day for tumor growth and animals euthanized when tumors reached 15 mm in any direction. **a** Response of individual tumors to treatment. Data is displayed as percent tumor area (LxW) compared to tumor area immediately prior to initiation of treatment. **b** Tumor area (LxW) in individual mice at day 20 post tumor cell injection. Statistical significance was determined using unpaired students T-Test (* < 0.05, *** < 0.01). **c** Overall survival animal survival. Data represents summation of two individual experiments

We next asked how our PDL1 chimeric tumors would respond to OV with the hypothesis that they would either separate into discrete PDL1^+^ and PDL1^−^ groups or display a more linear dose response. Tumors generated from injection of either a 1:1 or 1:9 mixture of B16/F10^scramble^ and B16/F10^PDL1−/−^ cells were established for 7 days and then treated with MYXV. Tumors established from injection of pure B16/F10^scramble^ or B16/F10^PDL1−/−^ cells were also included as controls. Consistent with previous results treatment of fully PDL1 deficient tumors resulted in significant tumor regression (80%, 12/15 mice) while treatment of fully PDL1 capable tumors resulted in only short term stable disease. Treatment of both 1:1 and 1:9 chimeric tumors resulted in intermediate phenotypes with some animals displaying tumor regression and others displayed stable disease followed by relapse. When the outcomes of all animals were taken into account, the overall therapeutic efficacy of MYXV treatment correlated strongly with the percentage of PDL1 capable cells found in a tumor at the time of treatment with 1:1 chimeric tumors responding better then fully PDL1 capable tumors but not as well as 1:9 chimeric tumors (Figs 2a - c). These data suggest that tumors expressing PDL1 in a limited number of tumor cells will likely relapse following OV, however, these tumors will still gain more benefit from therapy than tumors expressing PDL1 on a high percentage of malignant cells.

### Partially PDL1 positive tumors display specific immunoediting of PDL1^−/−^ cells

**Fig. 3:**
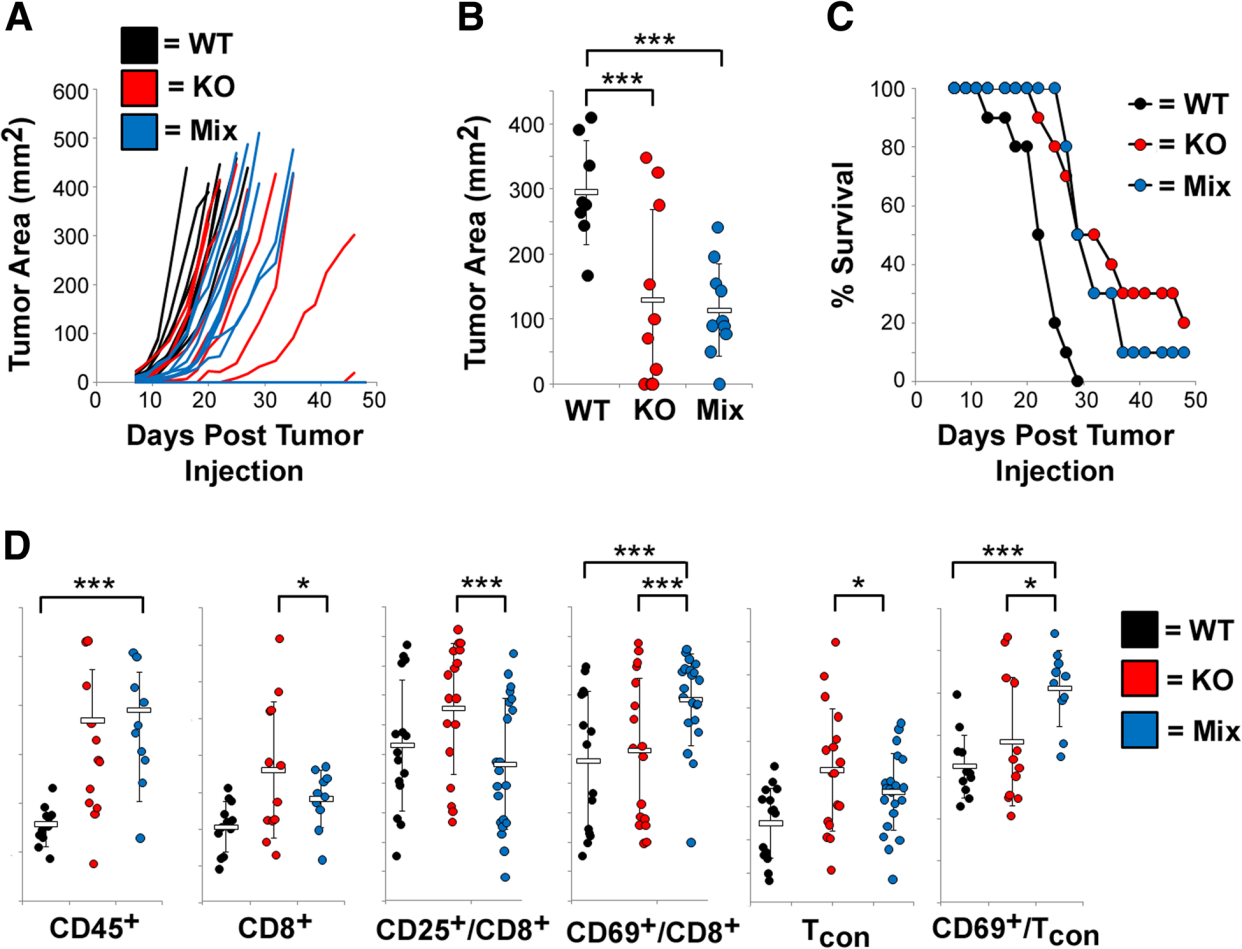
Phenotype of PDL1 Chimeric tumors. **a-c** 4 × 10^5^ total cells comprising pure cultures of either B16/F10^scramble^ (WT: *n* = 7) or B16/F10^PDL1−/−^ (KO: *n* = 9) or a 1:9 mix of WT to KO cells (Mix: *n* = 10) were injected SQ into the left flank of syngeneic C57/B6 mice. Tumors were then monitored every other day for tumor growth and animals euthanized when tumors reached 15 mm in any direction. **a** Tumor area (LxW) of individual tumors over time. **b** Tumor area (LxW) in individual mice at day 20 post tumor cell injection. Statistical significance was determined using unpaired students T-Test (*** < 0.01). **c** Overall animal survival. Data represents summation of two individual experiments. **d** C57/B6 mice were injected with WT (n = 15), KO (*n* = 17), or a 1:9 mix (*n* = 21) of cells as above. On day eight post injection, tumor cells were harvested, disassociated into single cells, and cellular composition analyzed using flow cytometry. Data represents summation of three independent experiments. Statistical significance was determined using unpaired students T-Test (* < 0.05,*** < 0.01)

To begin to understand why chimeric tumors displayed such reduced responsiveness to MYXV treatment, we next attempted to identify major differences between homogenous and mixed tumors before and after relapse. Phenotypically, chimeric tumors resulting from injection of a 1:9 ratio of B16/F10^scramble^ to B16/F10^PDL1−/−^ cells appeared to be similar to tumors generated from injection of pure B16/F10^PDL1−/−^ cells, displaying a slightly reduced growth rate compared to pure B16/F10^scramble^ tumors (Fig. 3a-c). Interestingly however, 8 days after implantation these chimeric tumors were immunologically distinct from both B16/F10^scramble^ and B16/F10^PDL1−/−^ tumors displaying the increased infiltration of total lymphocytes (CD45^+^ cells) seen in PDL1 deficient tumors but the reduced numbers of CD8^+^ and T_con_ cells typically seen in PDL1 capable tumors (Fig. 3d). Interestingly, both CD8^+^ and T_con_ cells found in chimeric tumors had also significantly increased expression of CD69 compared to both PDL1 deficient and capable tumors, suggesting that partial PDL1 positivity might influence both the immunological makeup and activity of tumors.

**Fig. 4:**
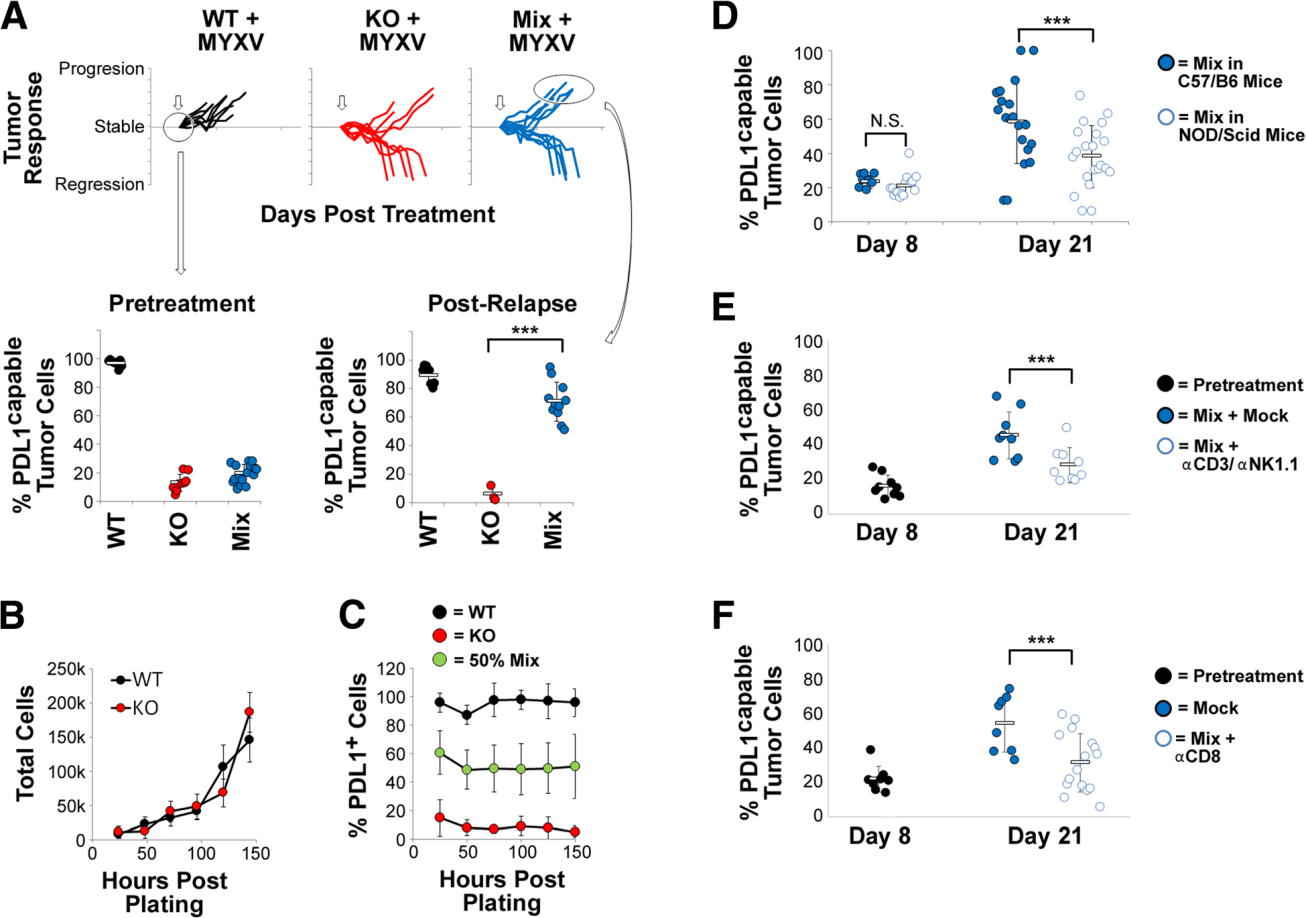
Relapsed PDL1 chimeric tumors display significant increases in PDL1 capable tumor cells. **a** 4 × 10^5^ total cells comprising pure cultures of either B16/F10^scramble^ (WT) or B16/F10^PDL1−/−^ (KO) or a 1:9 mix of WT to KO cells (Mix) were injected SQ into the left flank of syngeneic C57/B6 mice. Seven days post tumor implantation, tumors were treated with IT injection of either saline (Mock: WT n = 10, KO n = 10, Mix n = 10) or 1 × 10^7^ foci forming units of MYXV (MYXV: WT n = 9, KO n = 9, Mix n = 9). Treatment was repeated on days 9 and 11. Response of individual tumors to treatment. Data is displayed as percent tumor area (LxW) compared to tumor area immediately prior to initiation of treatment. The capability of tumor cells to express PDL1 was determined either immediately prior to therapy or at the time of euthanasia following relapse using flow cytometry. Data represents summation of two independent experiments. **b** Growth of WT or KO cells in vitro. Data represents summation of three independent experiments. **c** Equal numbers of WT, KO, or a 1:1 mix of each cell type (50% Mix) were plated in replicate wells of a 12 well dish. 12 h prior to harvest, cells were treated with IFNγ to upregulate PDL1 expression. At the indicated time points, cells were then harvested and the expression of PDL1 on the cell surface was analyzed using flow cytometry. Data represents summation of four independent experiments. **d** 4 × 10^5^ total cells comprising corresponding to a 1:9 mix of WT to KO cells were injected SQ into the left flank of either C57/B6 (black circles) or NOD/Scid (white circles) mice. Tumor makeup was analyzed either pre-treatment (day 8) or after MYXV therapy (day 21). Data represents the summation of two independent experiments. Significance was determined using students T test (*** < 0.01). **e** and **f** 4 × 10^5^ total cells comprising corresponding to a 1:9 mix of WT to KO cells were injected SQ into the left flank of C57/B6. A small number of animals were euthanized on day eight to analyze initial tumor makeup. Remaining mice were then separated into two cohorts and given MYXV treatment with either the indicated depleting antibodies (100μg/mouse once/week) or a control IgG (Mock). Tumor makeup was then analyzed following relapse (day 21)

Somewhat surprisingly, however, we found that while PDL1 capable, PDL1 deficient, and 1:9 chimeric tumors were immunologically distinct prior to oncolytic treatment, all of these tumor types displayed similar immunological signatures following OV (our unpublished observations). In contrast, analysis of the malignant cells found in each tumor before and after treatment revealed that relapsing chimeric tumors displayed a striking change in makeup. Consistent with our previous findings, immediately prior to the initiation of therapy 1:9 chimeric tumors were comprised of ~ 5–10% PDL1 capable cells. Following relapse, however, the percent of PDL1 capable cells found in these tumors had increased to comprise the vast majority of malignant cells recovered (Fig. 4a). This change in phenotype appeared specific to chimeric tumors, since tumors derived from pure cultures of either B16/F10^scramble^ or B16/F10^PDL1−/−^ cells retained their initial makeup following therapy. No obvious differences in the growth rates of B16/F10^scramble^ or B16/F10^PDL1−/−^ cells could be seen in vitro (Fig 4b and c) and both PDL1^+^ and PDL1^−/−^ cells were equally sensitive to MYXV infection (Additional file 1: Figure S3) suggesting that editing was not caused by differences in either inherent cell growth or sensitivity to viral infection. In contrast, editing occurred to a significantly lesser extent in immune deficient NOD/Scid mice (Fig. 4d) and could also be inhibited by depletion of either: CD3^+^ and NK1.1^+^ cells (Fig. 4e) or the specific CD8^+^ cytotoxic lymphocyte population (Fig. 4f). We therefore hypothesize that the change in phenotype seen in our mixed tumors following relapse represents a specific immunoediting of PDL1^−/−^ tumor cells.

## Discussion

An increasing number of studies have demonstrated that the PD1/PDL1 checkpoint pathway plays a significant role in determining the efficacy of OV [21–23, 25]. These studies, however, have used systemically injected PD1 blocking reagents to identify potential therapeutic synergies with oncolytic viruses and are therefore unable to address the specific impact of PDL1 expressed on various cell types. In contrast, in our current work we specifically eliminated PDL1 expression on malignant cells and demonstrate that tumors derived from these cells are highly susceptible to OV (Additional file 1: Figures S1 and S2). Interestingly, these tumors are not completely devoid of PDL1 as they contain relatively high numbers of CD45^+^/PDL1^+^ TIL (Fig. 3). These data therefore specifically implicate the PDL1 expressed on malignant cells as playing a highly significant role in the efficacy of OV. There are, however, several potential caveats to this conclusion. First, while our data clearly shows that tumors which fail to express PDL1 on malignant cells are susceptible to OV, it does not specifically demonstrate that the PDL1 expressed on TIL is not negatively impacting therapy. Indeed, it is possible that elimination of PDL1 from TIL would further enhance the efficacy of virotherapy. Alternatively, recent studies have shown that PDL1 expressed on either tumor cells or TIL can play significantly different roles in different models [26, 27]. Thus, it is possible that the high sensitivity of PDL1 deficient tumors to OV has to do with our use of the B16/F10 model. Further experiments are therefore needed to better clarify the role of tumor and TIL expressed PDL1 during OV.

The primary purpose of this work was to study the impact of partial PDL1 positivity on OV. To accomplish this, we utilized CRISPR/Cas9 technology to generate a matched set of PDL1 capable and PDL1 deficient cell lines which were then mixed ex vivo and injected into animals to form PDL1 chimeric tumors (Fig. 1). This methodology represents a relatively novel use of CRISPR technology which we feel has a number of significant uses in cancer research, including: loss of tumor antigens, mixed susceptibility to viral infection, mixed expression of tumor mutations, and dominance of various tumor phenotypes. Our studies, however, reveal several key factors which much be taken into consideration when conducting these types of studies. First is that the parent and daughter cell lines used must establish tumors with a linear correlation to the input mixture of cells. In our experiments, loss of PDL1 did not significantly impact the establishment of tumors in C57/B6 mice (Fig. 3). However, this is unlikely to be true for all proteins. If loss of a given protein significantly impacts tumor establishment, then the chimeric tumors resulting from injection of cell mixtures might have highly variable makeups which will complicate interpretation of results. Second is that it is highly beneficial if the cell lines used can be easily distinguished following tumor excision. For surface antigens, such as PDL1, this can be accomplished using flow cytometry, although care must be taken in identification of the tumor cells. Alternatively, tagging each cell line with a unique fluorophore (ie GFP vs RFP) might suffice, although care should be taken to account for differential immunogenicity if this approach is used.

Our results with this novel mixed tumor model demonstrate that even expression of PDL1 on a limited number of tumors cells can significantly impact the outcomes of therapy, causing relapse of 50–60% of tumors in the B16/F10 model (Fig. 2). Additionally, we found that the percentage of tumors cell capable of expressing PDL1 at the initiation of treatment displayed a strong inverse correlation with therapeutic outcome. Taken at face value, these data suggest that tumors expressing PDL1 in a limited number of tumor cells will likely relapse following OV, however, these tumors will still gain more benefit from therapy than tumors expressing PDL1 on a high percentage of malignant cells. This conclusion, however, is subject to a number of caveats. First and foremost is that our use of CRISPR/Cas9 to genetically ablate expression of PDL1 prevents the inducible expression of this protein which is frequently seen following OV [28–30]. Our results must therefore be interpreted more in the context of constitutive PDL1 expression and not the context of inducible expression. Additionally, our studies also suffer from the typical limitations associated with rapidly progressing injectable tumor models. In our case, the foremost of these limitations is the inability to study the long-term impact of partial PDL1 positivity on anti-tumor immunity. Clinical tumors displaying constitutive PDL1 expression will have months or years for this partial positivity to impact anti-tumor immune function. In contrast, treatment in our model must be initiated within 7–10 days of tumor establishment due to the extremely rapid tumor progression. Low level PDL1 expression within a partially positive tumor might therefore impact anti-tumor immunity in ways not seen in our current studies. Finally, our studies were unable to identify the morphological architecture of our partially PDL1 positive tumors and it therefore remains unclear how the PDL1^+^ and PDL1^−^ cells are distributed within each tumor. Critically, the nature of this distribution pattern might significantly influence the outcomes of treatment. Additional experiments are therefore required in order to more fully address this issue.

Interestingly, we observed that partially PDL1 positive tumors which relapsed following treatment displayed a significant increase in the percentage of PDL1 capable tumor cells (Fig. 4). This increase does not appear to be due to simple cellular growth rates since both the B16/F10^scramble^ and B16/F10^PDL1−/−^ cells used in our studies displayed identical growth rates (Fig. 4b) and selection for PDL1^+^ cells did not occur following mixed culture in vitro (Fig. 4c). Instead, our data suggests that this change in phenotype is due to active immunoediting and implicates CD8^+^ T cells in this process (Fig. 4). Interestingly, however, we routinely observed that loss of adaptive immunity reduced, but did not completely prevent, editing of our tumors (Fig. 4d-f). Therefore, while our data does implicate adaptive immune cells in this process, we cannot rule out other mechanisms such as intrinsic PDL1 survival signaling [31, 32]. Interestingly, previous work on intrinsic PDL1 signaling suggested that PDL1 deficient B16/F10 cells display an inherent growth defect in vitro compared to PDL1 capable cells, however, this defect was not observed in our studies (Fig. 4). The reason for this discrepancy is not clear; however, it has been shown that the intrinsic growth defects caused by ablation of PDL1 are amplified by both PD1 engagement and the presence of signaling molecules, such as interferon [31]. Neither of these factors was obviously present during our in vitro growth analysis which might explain the difference between our results and previous findings. Regardless, additional studies into the mechanism(s) mediating this editing and its impact on therapeutic outcomes therefore seem warranted.

## Methods

### Cell lines and reagents

B16/F10 (Cat# CRL-6475) and BSC40 cell lines (Cat# CRL-2761) were purchased from ATCC (Manassas, VA, USA). LLC and LLC-A9F1 cells were a kind gift form Dr. Mark Rubinstein. PDL1 deficient B16/F10 cells (B16/F10^PDL1−/−^) and control B16/F10 cells treated with a scrambled gRNA (GCGAGGTATTCGGCTCCGCG) (B16/F10^scramble^) were generated using the CRISPR/CAS9 system (Genscript, Piscataway, NJ, USA) as described previously [23]. MYXV (strain Lausanne) expressing GFP from an intergenic region between the *m135r* and *m136r* viral open reading frames (vGFP) has been described elsewhere [23]. Cell viability was determined using the CellTiter-96 Non-Radioactive Cell Proliferation Assay (Promega, Madison, WI, USA) per manufacturer’s recommendations. IFNγ was purchased from Sino Biological (Wayne, PA, USA) and used at a concentration which was functionally titered for each lot. The following antibodies were used in these studies. For flow cytometry: PDL1 (clone MIH5), PD1 (clone J43), CD3 (clone 145-2c11), CD4 (clone RM4–5), CD8 (clone 53–6.7), CD69 (clone H1.2F3), CD25 (clone 3C7), CD45 (clone 30-F11), CD44 (clone IM7), F4/80 (clone T45–2342), and Ly6c (clone AL21). For western blot: PDL1 (Cat# AF1019) (R&D Systems, Minneapolis, MN, USA), and actin (clone I19) (Santa Cruz Biotechnology, Dallas, TX, USA). For immune depletion: CD3 (clone 145-2C11), CD8 (clone 53–6.7), NK1.1 (Clone PK136) (BioXcell, West Lebanon, NH).

### In vivo tumor models

Six to eight week old C57/B6 (Charles River Laboratories, Raleigh, NC, USA) or NOD/Scid (NOD.CB17-Prkdc^scid^/J, The Jackson Laboratory, Bar Harbor, ME, USA) mice were injected subcutaneously (SQ) with 4 × 10^5^ total cells in 100ul low growth factor matrigel at a final concentration of 1.5 mg/ml (Corning, Corning, NY, USA). For mixed tumor studies, B16/F10^scramble^ and B16/F10^PDL1−/−^ cells were mixed at the indicated ratios ex vivo to give a total of 4 × 10^5^ cells and the mixtures injected as above. Viral treatment consisted of three intratumoral injections of either saline or vGFP (1 × 10^7^ total foci forming units (FFU) in 100ul PBS) on days 7, 9, and 11 post tumor implantation. For survival studies, tumor area was monitored using calipers and mice were euthanized when tumors reached 15 mm in any direction. For immunological and tumor analysis, tumors were excised, transferred onto a 40 μM nylon mesh filter and mechanically separated into a single cell suspension and then stained for flow cytometry using standard methodologies [23]. To upregulate PDL1 expression on cells capable of expressing this protein, tumor cells were incubated with IFNγ for 12 h prior to analysis. All analyses shown are pregated on single, viable events. All experiments were conducted in accordance with the Medical University of South Carolina Institutional Animal Care and Use Committee.

## Additional file

Additional file 1: **Figure S1.** B16/F10 tumors lacking PDL1 are highly susceptible to oncolytic immunotherapy. **Figure S2.** Lung cancer tumors naturally lacking PDL1 are highly susceptible to oncolytic therapy. **Figure S3.** Lack of PDL1 does not influence MYXV infection. (PDF 358 kb)
