# Supplementary material for: Chimeric tumor modeling reveals role of partial PDL1 expression in resistance to virally induced immunotherapy
Source: J Immunother Cancer. 2019 Jan 16;7:11. doi: 10.1186/s40425-018-0496-6 (PMC6335801; doi:10.1186/s40425-018-0496-6)
Supplement: Supplementary file 1 — Figure S1. B16/F10 tumors lacking PDL1 are highly susceptible to oncolytic immunotherapy. Figure S2. Lung cancer tumors naturally lacking PDL1 are highly susceptible to oncolytic therapy. Figure S3. Lack of PDL1 does not influence MYXV infection. (PDF 358 kb) [file 40425_2018_496_MOESM1_ESM.pdf]

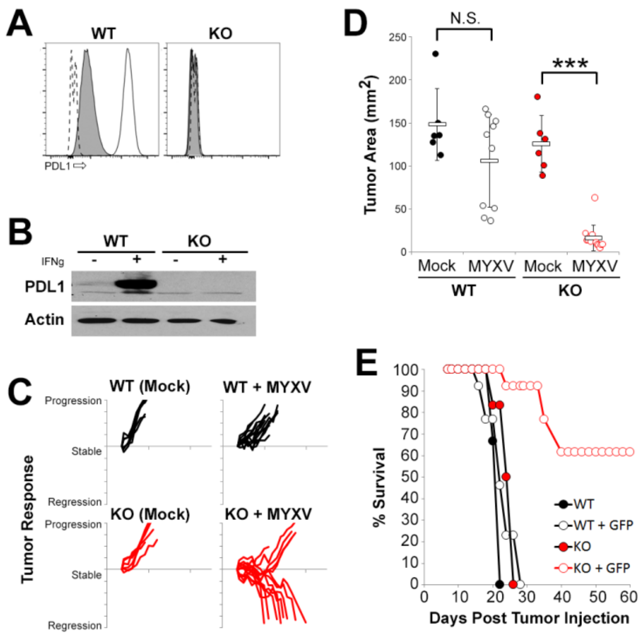


**Supplemental Figure 1 – B16/F10 tumors lacking PDL1 are highly susceptible to oncolytic immunotherapy. (A)** Expression of PDL1 was eliminated in B16/F10 cells using CRISPR/Cas9. A control cell line was also generated using a scrambled gRNA. Expression of cell surface PDL1 in either B16/F10$^{scramble}$ (WT) or B16/F10$^{PDL1-/-}$ (KO) cells analyzed using flowcytometry. Data is representative or more than three independent experiments. **(B)** Expression of PDL1 and Actin in whole cell lysates from either WT or KO cells analyzed by western blot. Data is representative of more than 3 independent experiments. **(C-E)** 4x10$^5$ WT (n=19) or KO (n=18) cells were injected SQ into the left flank of syngeneic C57/B6 mice. Seven days post tumor implantation, tumors were treated with IT injection of either saline (Mock: WT n=9, KO n=5) or 1x10$^7$ foci forming units of MYXV (MYXV: WT n=10, KO n=13). Treatment was repeated on days 9 and 11. Tumors were then monitored every other day for tumor growth and animals euthanized when tumors reached 15mm in any direction. **(C)** Response of individual tumors to treatment. Data is displayed as percent tumor area (LxW) compared to tumor area immediately prior to initiation of treatment. **(D)** Tumor area (LxW) in individual mice at day 20 post tumor cell injection. Statistical significance was determined using unpaired students T-Test (***<0.01). **(E)** Overall survival animal survival. Data is representative of more than three individual experiments.

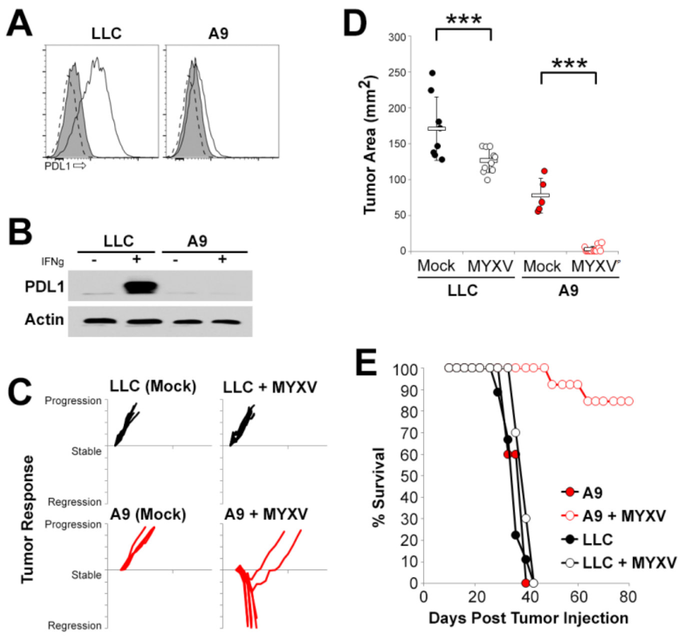


**Figure S2 – Lung cancer tumors naturally lacking PDL1 are highly susceptible to oncolytic therapy. (A)** Expression of cell surface PDL1 in either LLC or LLC-A9F1 (A9) cells analyzed using flowcytometry. Data is representative or more than three independent experiments. **(B)** Expression of PDL1 and Actin in whole cell lysates from either LLC or A9 cells analyzed by western blot. Data is representative of more than 3 independent experiments. **(C-E)** $4x10^5$ LLC (n=19) or A9 (n=18) cells were injected SQ into the left flank of syngeneic C57/B6 mice. Seven days post tumor implantation, tumors were treated with IT injection of either saline (Mock: LLC n=9, A9 n=5) or $1x10^7$ foci forming units of MYXV (MYXV: LLC n=10, A9 n=13). Treatment was repeated on days 9 and 11. Tumors were then monitored every other day for tumor growth and animals euthanized when tumors reached 15mm in any direction. **(C)** Response of individual tumors to treatment. Data is displayed as percent tumor area (LxW) compared to tumor area immediately prior to initiation of treatment. **(D)** Tumor area (LxW) in individual mice at day 20 post tumor cell injection. Statistical significance was determined using unpaired students T-Test (***<0.01). **(E)** Overall survival animal survival. Data is representative of more than three individual experiments.

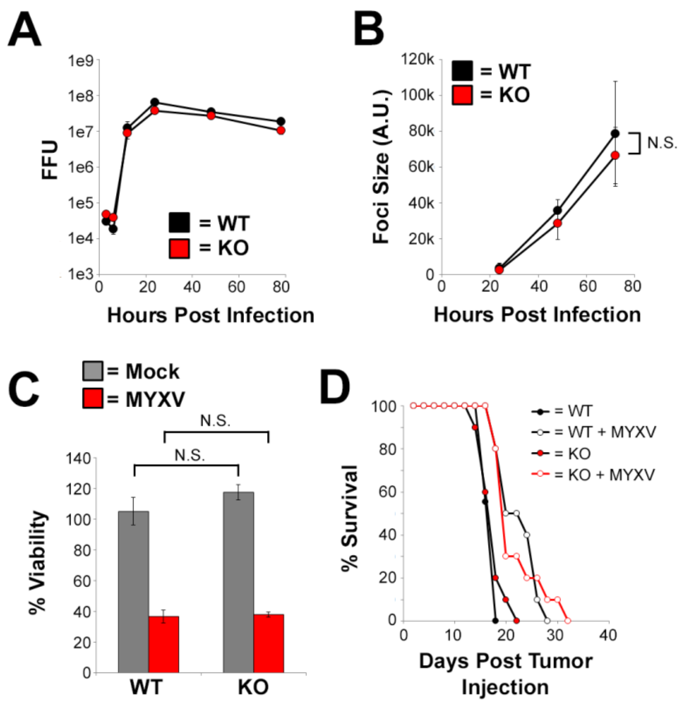


**Figure S3 – Lack of PDL1 does not influence MYXV infection. (A)** Intracellular production of new infectious MYXV progeny in either B16/F10$^{scramble}$ (WT) or B16/F10$^{PDL1-/-}$ (KO) cells measured using standard single step growth analysis. Data is representative of two independent experiments. **(B)** WT or KO cells were infected with MYXV at a low MOI. Pictures of GFP$^{+}$ foci were taken at the indicated time points and the size of individual viral foci was analyzed. Data is representative of two independent experiments and is displayed as average size of more than 15 foci at each time point (A.U. = arbitrary units). Statistical significance was determined using unpaired students T-Test. **(C)** Viability of either WT or KO cells 48 hours post infection with MYXV using MTT assay. Statistical significance was determined using unpaired students T-Test (N.S. = not significant). **(D)** 4x10$^{5}$ WT or KO cells were injected SQ into the left flank of immune deficient NOD/Scid mice. Seven days post tumor implantation, tumors were treated with IT injection of either saline (Mock: WT n=10, KO n=10) or 1x10$^{7}$ foci forming units of MYXV (MYXV: WT n=10, KO n=10). Tumors were then monitored every other day for tumor growth and animals euthanized when tumors reached 15mm in any direction. Data is representative of two independent experiments.
